# Cholecystectomy among Patients Admitted to the Department of Surgery in a Tertiary Care Centre: A Descriptive Cross-sectional Study

**DOI:** 10.31729/jnma.8198

**Published:** 2023-06-30

**Authors:** Kishor Kumar Deo, Sundar Shrestha, Aliska Niroula, Muna Khanal, Adarsha Kumar Jha, Shreya Niroula, Prajwala Yogi

**Affiliations:** 1 Department of Surgery, National Academy of Medical Sciences, Mahaboudha, Kathmandu, Nepal; 2 Kathmandu Medical College and Teaching Hospital, Sinamangal, Kathmandu, Nepal; 3 Nepal Medical College and Teaching Hospital, Jorpati, Kathmandu, Nepal; 4 Mahendra Narayan Nidhi Memorial Hospital, Kathmandu, Nepal

**Keywords:** *cholecystectomy*, *cholelithiasis*, *Nepal*, *prevalence*

## Abstract

**Introduction:**

Cholecystectomy refers to the surgical removal of the gallbladder. It is indicated in acute cholecystitis, and other complications of gallstones like cholecystitis, pancreatitis and bile duct obstruction, the presence of gallbladder trauma, and gallbladder cancer. The aim of this study was to find out the prevalence of cholecystectomy among patients admitted to the Department of Surgery in a tertiary care centre.

**Methods:**

A descriptive cross-sectional study was conducted among patients admitted to the Department of Surgery of a tertiary care centre. Data from 1 July 2021 to 1 July 2022 were collected between 10 February 2023 to 20 February 2023 from the hospital records. Ethical approval was taken from the Institutional Review Committee of the same institute (Reference number: 630/2079/80). Convenience sampling method was used. Documents and medical records of the cholecystectomy patients were assessed for personal data, medical history was extracted and analysed using Microsoft Excel 2016. Point estimate and 95% Confidence Interval were calculated.

**Results:**

Among 2452 patients admitted to the Department of Surgery, 894 (36.46%) (34.46-38.26, 95% Confidence Interval) underwent cholecystectomy.

**Conclusions:**

The prevalence of cholecystectomy in our study was found to be similar to other studies done in similar settings.

## INTRODUCTION

Cholecystectomy refers to the surgical removal of the gallbladder.^1^ It is indicated in acute cholecystitis, and other complications of gallstones like cholecystitis, pancreatitis and bile duct obstruction, the presence of gallbladder trauma, and gallbladder cancer.^2^

Symptomatic cholelithiasis is one of the most common presenting complaints among patients with acute abdomen to the emergency department.^3,4^ It can result in various other conditions such as cholecystitis, pancreatitis, obstruction of the biliary tract and gallbladder cancer, leading to a significant medical burden.^4^ The prevalence of cholelithiasis differs not p>only between countries but also among different ethnic groups, age groups, and genders, differences in dietary and lifestyle patterns with a preference for junk food and sedentary lifestyles, and the incidence of gallstones is rising globally.^4,5^

Since there are very few studies conducted in Nepal regarding the prevalence of cholecystectomy among the patients admitted to the Department of Surgery, this article will be helpful in finding out the data. Hence, the aim of this study was to find out the prevalence of cholecystectomy among patients admitted to the Department of Surgery in a tertiary care centre.

## METHODS

This descriptive cross-sectional study was conducted among the patients admitted to the Department of Surgery of the National Academy of Medical Sciences, Bir Hospital, Nepal. Data from 1 July 2021 to 1 July 2022 were collected between 10 February 2023 to 20 February 2023 from the hospital records. Ethical approval was taken from the Institutional Review Committee of the same institute (Reference number: 630/2079/80 ). All the patients admitted to the Department of Surgery with complete hospital record data were included in this study. Patients with incomplete hospital record data and illegible handwriting were excluded from the study. Convenience sampling method was used. The sample size was calculated using the following formula:


$$\begin{array}{ll} \text{n} & ={\text{Z}}^{2}\times \frac{\text{p}\times \text{q}}{{\text{e}}^{\text{2}}}\\ & =1.96^{2}\times \frac{0.50\times 0.50}{0.03^{2}}\\ & =1068 \end{array}$$

Where,

n = minimum required sample size
  Z = 1.96 at 95 % Confidence Interval
  p = prevalence taken as 50% for maximum sample size
q = 1-p
e = margin of error, 3%

The minimum required sample size was 1068. Doubling the sample size, we get 2136. However, the final sample size taken was 2452.

Documents and medical records of the cholecystectomy patients were assessed for personal data, and medical history.

Data were analysed using Microsoft Excel 2016. Point estimate and 95% CI were calculated.

## RESULTS

Among 2452 patients admitted to the Department of Surgery, 894 (36.46%) (34.46-38.26, 95% CI) underwent cholecystectomy. The mean age of patients who underwent cholecystectomy was 44.55±14.49 years. A total of 837 (93.62%) patients underwent cholecystectomy which was indicated for symptomatic cholelithiasis (Table 1).

**Table 1:** Indications of cholecystectomy (n= 894).

Indication of cholecystectomy	n (%)
Symptomatic cholelithiasis	837 (93.62)
Acute calculus cholecystitis	29 (3.24)
Gall bladder polyp	11 (1.23)
Acute biliary pancreatitis	4 (0.45)
Carcinoma of gall bladder	4 (0.45)
Chronic calculus cholecystitis	3 (0.34)
Acute cholangitis with acute mild cholecystitis with cholelithiasis	1 (0.11)
Acute mild calculus cholangitis	1 (0.11)
Asymptomatic cholelithiasis	1 (0.11)
Cholangitis with choledochal cyst	1 (0.11)
Choledocholithiasis	1 (0.11)
Gall bladder sludge	1 (0.11)

A total of 48 (5.73%) patients with symptomatic cholelithiasis presented with comorbidities. Among them, 21 (43.75%) patients had hypertension (Table 2).

**Table 2:** Symptomatic cholelithiasis with comorbidities (n= 48).

Comorbidities	n (%)
Hypertension	21 (43.75)
Diabetes mellitus	6 (12.50)
Hypothyroidism	4 (8.33)
Hypertension with diabetes mellitus	5 (10.41)
Hypertension with hypothyroidism	2 (4.17)
Hypertension with depression	1 (2.08)
Chronic obstructive pulmonary disease	1 (2.08)
Duodenal polyp	1 (2.08)
Biliary pancreatitis	1 (2.08)
Hereditary spherocytosis	1 (2.08)
Umbilical hernia	2 (4.17)
Incisional hernia	1 (2.08)
Right inguinal hernia	1 (2.08)
Subxiphoid hernia	1 (2.08)

Majority of patients 716 (80.09%) were female and 178 (19.91%) were male (Table 3).

**Table 3:** Gender of patients who underwent cholecystectomy (n= 894).

Gender	n (%)
Female	716 (80.09)
Male	178 (19.91)

## DISCUSSION

The most frequent biliary pathology is gallstones, and cholecystectomy is one of the procedures that general surgeons undertake the most frequently. There are clear relationships between gallstone production and age, sex, heredity, obesity, food, and infection, according to studies on a variety of causative factors in various populations.^6^

The prevalence of cholecystectomy in our study is 36.34% which is more in comparison to other studies done in similar settings.^7^ The mean age of patients with cholecystectomy in our study was 44.55±14.49 years. Many studies have shown that age is an established risk factor for gallstones, with the incidence progressively increasing each decade.^8,9^

The most common indication of cholecystectomy was symptomatic cholelithiasis with a prevalence of 93.62%. Similar results are shown in other studies done in Florida.^2^ About 80.09% of the patients who underwent cholecystectomy were female which is in accordance with the study done in South India and North India.^3,4^ Our study showed that 5.73% of patients with symptomatic cholelithiasis presented with comorbidities among which 43.75% of patients had hypertension but a study done in India had the highest prevalence in patients with hypertension and diabetes.^10^

Since the study is single institution-based and a descriptive cross-sectional, the results can not be generalised in the larger population. Also, a study design with a higher level of evidence is recommended for future studies.

## CONCLUSIONS

The prevalence of cholecystectomy in our study is similar to other studies in similar settings. Since our study showed that most of were patients were female in their mid-forties with hypertension and diabetes, an analytical study with association to gender, age and co-morbidities should be conducted.
